# 15-hydroxyeicosatetraenoic acid (15-HETE)-GPR39 axis: a potential therapeutic target in eosinophilic asthma

**DOI:** 10.3389/fphar.2026.1880829

**Published:** 2026-10-06

**Authors:** Andrew Engel, Zhenying Nie, Sanjiv Kaul

**Affiliations:** 1 Knight Cardiovascular Institute, Oregon Health and Science University, Portland, OR, United States; 2 Division of Pulmonary, Allergy and Critical Care Medicine, Oregon Health and Science University, Portland, OR, United States

**Keywords:** 15-hydroxyeicosatetraenoic acid, 15-lipoxygenase, arachidonic acid, eosinophilic asthma, GPR39

## Abstract

Arachidonic acid (AA) metabolites play key roles in the pathophysiology of asthma. Along with cyclooxygenase and CYP P450, lipoxygenases (LO) produce these metabolites. 15-hydroxyeicosatetraenoic acid (15-HETE), a product of 15-LO, has been implicated in severe asthma, particularly eosinophilic asthma. This article reviews the AA–15-HETE axis in eosinophilic asthma. 15-HETE contributes to key features of eosinophilic asthma, including eosinophil recruitment, mucus hypersecretion, and airway remodeling. Despite these observations, the downstream signaling mechanisms through which 15-HETE exerts its effects remain incompletely understood. GPR39 has recently been proposed as a potential receptor for 15-HETE, linking lipid metabolism to intracellular signaling involved in inflammation and tissue remodeling. Although the role of GPR39 in asthma has not been studied, its ability to respond to bioactive lipids suggests it may serve as a critical signaling node in eosinophilic inflammation. Taken together, these findings support a model in which the AA–15-HETE–GPR39 axis contributes to the pathogenesis of eosinophilic asthma. Targeting GPR39 may therefore represent a novel and underexplored therapeutic strategy for modulating airway inflammation and remodeling.

## 1 Introduction/background

Asthma is a chronic inflammatory airway disease characterized by wheezing, chest tightness, coughing, and shortness of breath. It is caused by airway narrowing resulting from bronchoconstriction, airway hyperresponsiveness, increased mucous production, and structural remodeling of the airway wall (Hussain and Liu, 2024). Asthma prevalence in the United States has been rising (Pate and Zahran, 2024). In 2024, 8.6% of adults and 6.5% of children were affected (Prevention, 2024). Diagnosis of asthma requires demonstration of an increase in forced expiratory volume at 1 s (FEV_1_) of ≥12% and ≥200 mL from baseline following inhaled bronchodilator administration (Levy et al., 2023). Various triggers, including viral infection, allergens, strong smells, smoke, and exhaust fumes, as well as exercise and laughter can precipitate or exacerbate asthmatic attacks (World Health Organization, 2026). The chronic effects of asthma lead to complications, increase healthcare costs, and decrease quality of life (Pate and Zahran, 2024). While most patients with symptoms are controlled with standard therapy, a subset develops severe asthma that requires additional medications, including inhaled corticosteroids (Levy et al., 2023).

Eosinophilic asthma accounts for approximately two-thirds of patients with severe asthma and is notable for eosinophilic infiltration of the lung, Th2 cytokine activity, and risk of frequent exacerbation (Hussain and Liu, 2024; Denton et al., 2021; Porpodis et al., 2022). In this condition, eosinophils are activated and release inflammatory mediators derived from arachidonic acid (AA), such as leukotrienes (Figure 1), which contribute to airway inflammation and bronchoconstriction. While leukotrienes have been extensively studied in asthma, increasing attention has turned to 15-hydroxyeicosatetraenoic acid (15-HETE), an AA metabolite (Figure 1) in the pathogenesis of asthma (Kytikova et al., 2024; Shuaib Nasser and Lee, 2002). 15-HETE in this article refers to 15(S)-HETE and not its stereoisomer 15(R)-HETE. This review examines the immunopathogenesis of the AA–15-HETE axis in eosinophilic asthma and discusses the potential effects of targeting GPR39, the receptor for 15-HETE (Alkayed et al., 2022), as a novel therapeutic strategy.

**FIGURE 1:**
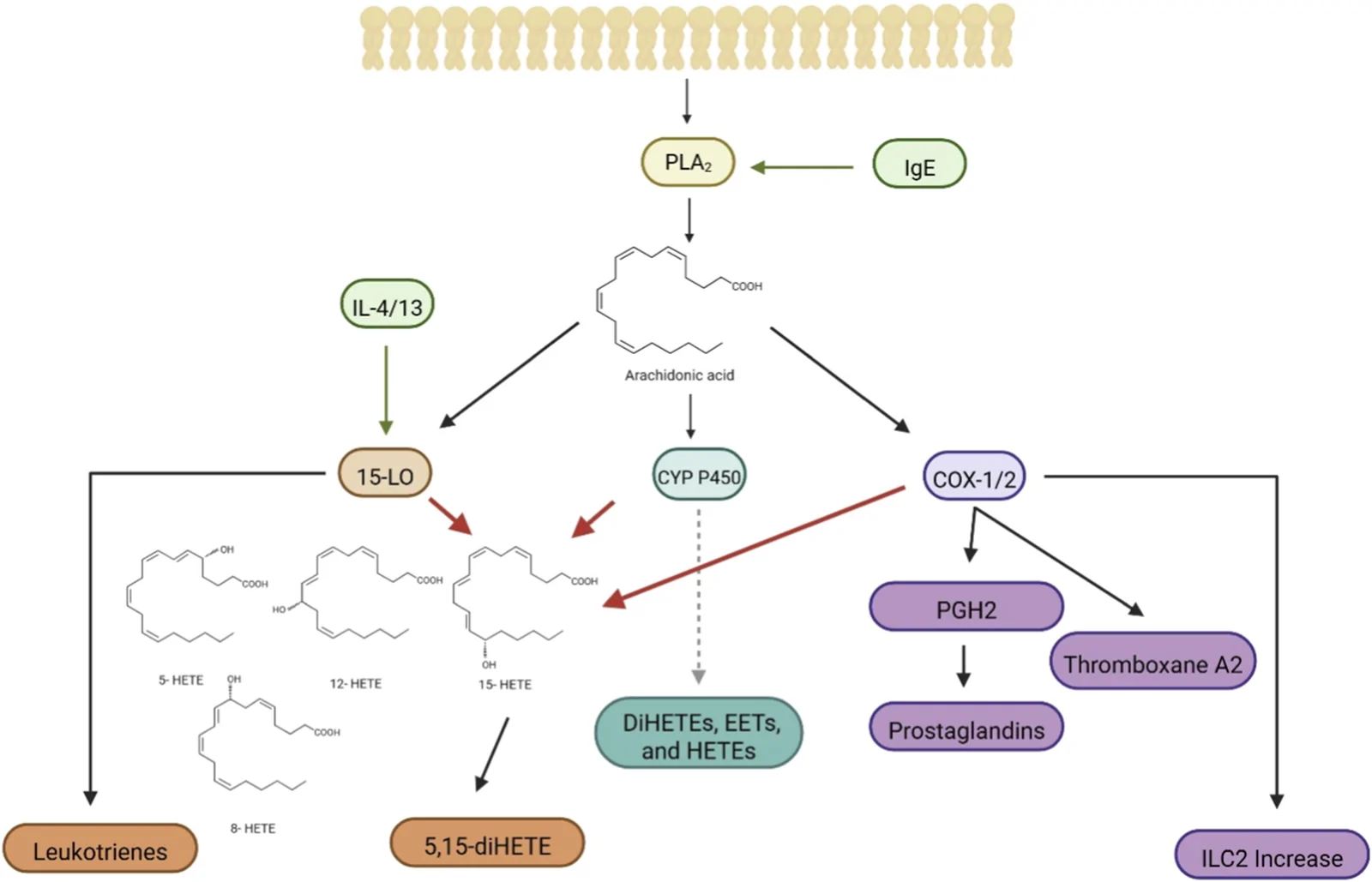
AA (AA) metabolites. After release from plasma membrane by PLA_2,_ AA can be metabolized by 3 enzymatic systems: Cytochrome (CYP) 450, cyclooxygenasess, and lipoxygenases. The CYP 450 pathway further involves 3 sets of enzymatic systems: epoxygenases, hydroxylases, and ώ-hydroxylases. Although 15-HETE is predominantly produced by lipoxygenases, it can also be produced by other enzymatic pathways as shown by the red arrows. PLA_2_ can be activated by IgE. ILC2 = Group 2 innate lymphoid cells. Created in https://BioRender.com.

## 2 Immunopathogenesis of eosinophilic severe asthma

Pathogenesis of severe asthma is heterogeneous and categorized into eosinophilic and non-eosinophilic subtypes, with eosinophilic asthma accounting for the majority of all severe cases (Hussain and Liu, 2024; Denton et al., 2021; Porpodis et al., 2022). Eosinophilic asthma is characterized by elevated peripheral blood eosinophil count (>150 cells/µL) that can exceed 400 cells/µL (Skolnik and Carnahan, 2019; Corren et al., 2021). Increased eosinophil levels are also observed in sputum and bronchial biopsies (Zhang and Wenzel, 2007; de Groot et al., 2015). Eosinophilic asthma is driven by Type 2 inflammation, with hallmark increases in IL-4, IL-5, and IL-13 expression, which promote eosinophil recruitment, activation, and survival (Carr et al., 2016; Wenzel et al., 1999). It is also associated with higher levels of CD3, CD4, CD8, and CD68 lymphocytes, as well as mast cell and increased transforming growth factor-β (TGFβ) expression, reflecting both inflammation and airway remodeling (Carr et al., 2016).

Beyond cytokines and chemokines, lipid mediators produced by eosinophils have emerged as important modulators of eosinophilic inflammation. Among these, 15-HETE (Figure 1) has received increasing attention for its potential roles in promoting airway inflammation, mucus hypersecretion, and epithelial cell remodeling (Porpodis et al., 2022; Kytikova et al., 2024). 15-HETE may serve as a critical link between type 2 immune activation and eosinophilic infiltration in severe asthma (Endo et al., 2014).

## 3 15-HETE in asthma pathology

### 3.1 Biosynthesis of 15-HETE in eosinophilic asthma

15-HETE is a bioactive metabolite of AA, generated primarily via 15-lipoxygenase (15-LO) activity (Figure 1) (Porpodis et al., 2022; Kytikova et al., 2024). AA is released from cell membranes by phospholipase A_2_ (PLA_2_) (Figure 1) expressed in pulmonary epithelial cells, macrophages, and eosinophils (Nolin et al., 2019). PLA_2,_ levels are negatively correlated with FEV_1_ values and asthma severity (Bowton et al., 1997). Both levels of PLA_2_ and its substrate AA are increased by antigen challenge in asthma patients (Pniewska and Pawliczak, 2013; Hallstrand et al., 2013), leading to increased substrate availability for 15-LO–mediated 15-HETE production (Figure 1). PLA_2_ activity can also be promoted by IgE activation of airway mast cells, leading to increased AA release and subsequent 15-HETE generation (Hamu-Tanoue et al., 2024) (Figure 1).

AA can be preferentially metabolized via a specific pathway by certain triggers (Zhao et al., 2017). For instance, Th2 positive asthmatics experience higher 15-HETE levels due to induction of 15-LO compared to other enzymes, shifting metabolism of AA towards increased 15-HETE rather than other eicosanoids or prostaglandins (Jayawickreme et al., 1999). This effect occurs primarily in pulmonary epithelial cells and eosinophils (Brown et al., 2001). The increased production of 15-HETE, like increased 15-LO and Th2 activity, occurs specifically in severe eosinophilic patients.

15-LO is necessary for 15-HETE formation (Jayawickreme et al., 1999). As expected, increasing both AA availability and 15-LO incubation time enhances 15-HETE production (Brown et al., 2001; Profita et al., 2000). In cultured airway cells, interleukin signaling can induce 15-HETE production without changes in AA concentrations (Zhao et al., 2011; Spanbroek et al., 2001; Gulliksson et al., 2007; James et al., 2013), indicating that 15-HETE levels are controlled by both enzymatic activity and immune-mediated regulation.

### 3.2 Role of 15-lipoxygenase in eosinophilic asthma

15-LO is a key enzyme in the generation of 15-HETE from AA (Figure 1). In humans it is encoded by *ALOX15* (Singh and Rao, 2019; Kuhn et al., 1990) and primarily oxygenates AA at the 15 carbon position to produce 15-HETE, but can also oxygenate AA at the 5th and 12th, carbon positions, creating 5-, and 12-HETE, respectively (Kuhn et al., 1990). Two major isoforms exist: 15-LO-1 (also referred to as 15-LOA) and 15-LO-2 (also referred to as 15-LOB) (Gan et al., 1997; Brash et al., 1997). While 15-LO-1 preferentially produces 15-HETE, it can also generate other HETEs. In comparison 15-LO-2 is selective for 15-HETE production only.

Under physiological conditions, 15-LO is constitutively expressed predominantly in the tracheal epithelium, leukocytes, and perineural cells, and to a lesser extent in the submucosa, blood vessels, and airway smooth muscle (Holtzman, 1992; Ivanov et al., 2015). 15-LO plays an important role in late stage of epithelial cell development, being upregulated after mucous differentiation in the airway epithelium (Hill et al., 1998). In asthma, 15-LO expression and activity are increased in airway tissues, particularly in moderate and severe disease. Elevated 15-LO-1 has been detected in the bronchial epithelium and submucosa of asthmatics; induced sputum from asthmatic patients also exhibits upregulation of 15-LO mRNA in macrophages (Zhao et al., 2009). 15-LO activity is also higher in bronchoalveolar lavage (BALF) of eosinophilic positive compared to eosinophilic negative patients. In contrast, a study reported no difference in 15-LO-1 expression in any grade of asthma compared to controls (Wenzel et al., 1999). Within circulating blood cells of normal individuals, 15-LO-1 is mainly expressed in macrophages and eosinophils. In atopic asthmatics, 15-LO expression is increased in leukocytes but not in mononuclear cells (Sridhar et al., 2019).

It is worth noting that 15-LO measurement are influenced by the specific assay used. In some cases, only 15-LO-1 is measured despite 15-LO-2 being more selective at producing 15-HETE (Shannon et al., 1991). Whereas 15-LO-2 is also independently upregulated in both blood and lungs of severe asthmatics (Shannon et al., 1991), it is sometimes included in the analyses although, more often excluded or not specified.

Deletion of 15-LO decreases asthmatic presentation in animal models (Hajek et al., 2008). 12/15-LO, the murine ortholog to human 15-LO-1, is needed in the lung to develop a response after inhaled allergen. Ovalbumin sensitized 12/15 LO knockout mice do not develop asthma when the antigen is inhaled (Andersson et al., 2008). However, intraperitoneal injection of ovalbumin still produces a characteristic asthmatic response in the lung (Chang et al., 2005), indicating the need of local 12/15LO activity in the lung to induce inhaled allergen induced asthma.

Eosinophilic asthma is a classic manifestation of a Th2 immune response, in which CD4^+^ Th2 cells produce cytokines such as IL-4, IL-5, and IL-13 that drive IgE production followed by eosinophil recruitment, activation, and survival in the airways (Figure 2) (Shankar et al., 2022). IL-4 mainly upregulates 15-LO-1 but can also stimulate 15-LO-2 production, and participates in 15-HETE incorporation into epithelial cells (Profita et al., 2000). IL-4 plays a larger role than IL-13 in the blood inflammatory response (Shankar et al., 2022), upregulating 15-LO1 in dendritic cells (Zhao et al., 2011) and mast cells derived from human cord blood (Gulliksson et al., 2007). In contrast, IL-13’s role is more local to the pulmonary epithelium and bronchial smooth muscle, where it stimulates 15-HETE production via 15-LO-1 and not 15-LO-2 activation (Sridhar et al., 2019). These cytokines promote increased 15-HETE production and link Th2 immune activation directly to altered AA metabolism (Brunnstrom et al., 2015).

**FIGURE 2:**
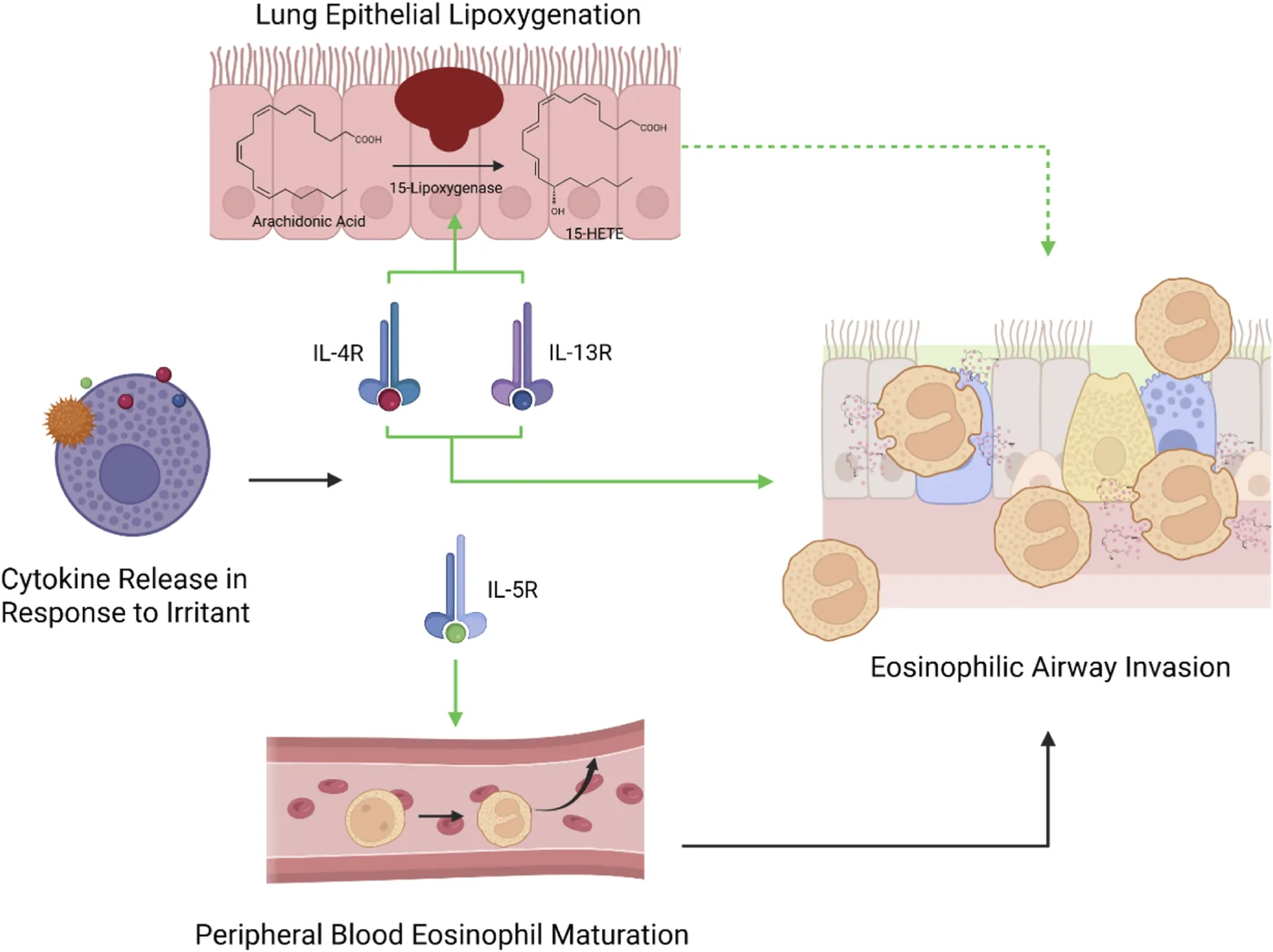
Dual Influence of IL-4 and IL-13 in the airway. In Th2 individuals, the inflammatory response releases IL-4, IL-5, and IL-13. IL-5 circulates within the blood, inducing eosinophil maturation. Following cytokine release, IL-4 and IL-13 initiate two separate pathways within the lung. The first induces 15-Lipoxygenase within epithelial cells, and the second traffics eosinophils into the airway. 15-HETE is responsible for eosinophilic invasion. Created in https://BioRender.com.

Collectively, these findings indicate that 15-LO is upregulated in eosinophilic asthma, particularly in severe and Th2-high phenotypes, where it contributes to increased 15-HETE production and eosinophilic airway inflammation.

#### 3.2.1 Paradoxical activities of 15-LO in asthma

12/15-LO is not only involved in the pathogenesis of asthma, but it is also involved in its resolution (Miyata et al., 2013; Ito et al., 2025; Miyata et al., 2026). It produces anti-inflammatory lipid metabolites, collectively termed specialized pro-resolving mediators from eosinophils and pleural macrophages. These mediators are found in later stages of disease and can suppress eosinophilic inflammation. Lower 15-LO levels result in less of these mediators, which are associated with more severe asthma in humans. These mediators reduce inflammation when administered after allergen challenge in mice. 15-HETE can be metabolized to eoxins, a pro-resolving mediator, through the biosynthetic pathway of cysteinyl leukotriene. 12/15-LO also metabolizes the omega-3 fatty acids, eicosapentaenoic and docosahexaenoic acids into pro-resolving mediators. Thus, the role of 12/15 LO is more nuanced than simply being a pro-inflammatory mediator.

#### 3.2.2 Role of cyclooxygenases in eosinophilic asthma

Cyclooxygenases (COX-1 and COX-2) can also contribute to 15-HETE production from AA (Figure 1) (Miyata et al., 2013). COX-1 is highly expressed in mast cells where it is involved in AA derived lipid synthesis (Kowalski et al., 2007). IgE-mediated mast cell activation promotes AA release and can stimulate 15-HETE production (Kowalski et al., 2007). In aspirin exacerbated respiratory disease (AERD), increased mast cell density and COX-1 activity are associated with elevated 15-HETE levels, and COX-1 inhibition reduces 15-HETE generation in mast cells (Kowalski et al., 2003). However, the relevance of COX-mediated 15-HETE production in eosinophilic severe asthma appears limited. In atopic and eosinophilic asthma, both mast cells and eosinophils demonstrate increased 15-LO activity (Gulliksson et al., 2007; Sridhar et al., 2019), whereas COX upregulation is less consistently associated with disease severity. In house dust mite models, BALF samples do not demonstrate significant increase in mast cells and both 12/15-LO and 5-LO are upregulated more than either COX enzyme (Kumlin et al., 1991).

Pharmacologic evidence further supports this distinction. Although COX-1 inhibitors such as aspirin reduce COX-dependent 15-HETE production, total 15-HETE levels in eosinophilic asthma are largely maintained through 15-LO activity (Kumlin et al., 1991). Thus, while COX enzymes can contribute to 15-HETE generation under certain conditions, particularly in AERD, 15-LO represents the predominant and disease-relevant pathway in eosinophilic severe asthma.

### 3.3 Role of 15-HETE in eosinophilic asthma

#### 3.3.1 Elevated 15-HETE in asthma

15-HETE is constitutively produced in the airway epithelium and dynamically regulated under both basal and stress conditions. Both asthmatic and non-asthmatic lungs convert AA to 15-HETE at the same rate in culture (Alpert and Walenga, 1993). In explanted healthy human lung tissue, bronchi produce 15-HETE when exposed to excess AA and calcium ionophore (Alpert and Walenga, 1993). Intracellularly, 15-HETE availability is controlled through esterification and reincorporation into membrane phospholipids-particularly phosphatidylinositol in tracheal epithelial cells (Salari and Chan-Yeung, 1989; Kolmert et al., 2018). This reflects both basal intracellular production and inflammation-induced release of 15-HETE. Environmental stressors such as ozone increase epithelial reincorporation of 15-HETE (Alpert and Walenga, 1995).

15-HETE levels are elevated in the lungs of patients with severe, particularly eosinophilic, asthma (Turi et al., 2023). Pulmonary tissue and BALF from severe asthmatics contain higher levels of 15-HETE compared to mild asthmatics and healthy controls. Similar increases are observed in house dust treated mice (Turi et al., 2023). Importantly, increased 15-HETE production in severe asthmatics appears specific to eosinophilic positive patients (Chu et al., 2002). Although tissue levels of 15-HETE are increased, the amount of exhaled 15-HETE may decrease in asthmatics (Chen et al., 2021), likely due to its reincorporation within cell membranes.

15-HETE production is closely associated with IgE-mediated inflammatory responses such as Th2 inflammation and aspirin-sensitive reactions (Luu et al., 2022). Cultured human airway cells release more 15-HETE following IgE stimulation when AA is available, whereas IgE exposure alone does not increase 15-HETE production (Turi et al., 2023). Elevated 15-HETE has also been linked with Th2-associated chemokines such as thymus and activation regulated chemokine (TARC), which is high in asthmatics (Luu et al., 2022). In aspirin sensitive asthmatics, peripheral blood leukocytes show increased 15-HETE levels following an aspirin challenge, a response not observed in aspirin tolerant asthmatics (Skevaki et al., 2025).

Aspirin sensitivity amplifies 15-HETE responses in airway tissues. *In vitro* aspirin exposure increases 15-HETE production in lung samples from aspirin sensitive asthmatics but not aspirin tolerant subjects (James et al., 2013; Shankar et al., 2022; Brunnstrom et al., 2015). Airway epithelial cells from aspirin sensitive group exhibit increased 15-HETE levels following aspirin challenge despite similar baseline levels (Jedrzejczak-Cz et al., 2008; Lewandowska-Polak et al., 2011). Notably, these aspirin-induced increases in 15-HETE occur without parallel elevations in other inflammatory mediators such as leukotriene C_4_, eosinophil cation protein, or tryptase (Kowalski et al., 2000).

Elevation of 15-HETE in asthma is localized to pulmonary tissues rather than the systemic circulation. Whereas 15-HETE levels are increased in eosinophils, there is no difference in 15-HETE levels across all leukocytes between healthy and asthmatic subjects (Kowalski et al., 2005). Similarly, 15-HETE levels in the urine and serum of asthmatics remain unchanged (Ban et al., 2021), indicating that 15-HETE dysregulation is largely compartmentalized to the lung. In NSAID-sensitive asthma, both 15-HETE and its metabolite 15-oxo-ETE (Szatkowski et al., 2024), are increased; because 15-oxo-ETE is a potent eosinophil chemoattractant derived from 15-HETE (Powell and Rokach, 2013), this indirectly implicates 15-HETE in tissue eosinophil recruitment.

Baseline 15-HETE levels may influence disease phenotype and therapeutic response in patients with AERD who exhibit higher baseline exhaled 15-HETE levels than aspirin tolerant patients and experience a greater decrease in exhaled 15-HETE following aspirin challenge (Marom et al., 1983). In patients with AERD, higher baseline plasma 15-HETE levels are associated with greater symptomatic improvement following treatment (Mastalerz et al., 2012). Interestingly, individuals with higher baseline 15-HETE tend not to exhibit further increases upon challenge, whereas those with lower baseline levels show greater inducible increases and poorer symptom improvement (Mastalerz et al., 2012; Jerschow et al., 2017).

Collectively, these findings suggest that elevated 15-HETE levels in the lung contribute to the pathobiology of severe eosinophilic asthma.

#### 3.3.2 15-HETE induced lung changes-mucous secretion

15-HETE modulates airway mucus secretion, although its effects vary by concentration and species. In cultured human airway 15-HETE released mucous glycoproteins (Sridhar et al., 2019) at concentrations of 10 and 100 nM concentrations; however it did not increase mucus release in normal human tracheobronchial epithelial cells at micromolar concentrations (Chu et al., 2002). Compared with 15-HETE, 12-HETE induces greater mucus production in cultured human airway cells (Marom et al., 1983). In animal models, 15-HETE increases airway secretions more consistently: in dogs, both intraarterial injection and inhalation of 15-HETE increases fluid exudation and tracheal mucus; in rats intravenous injection of 15-HETE increases the mucous gel layer thickness - an effect reported to be more potent than induced by 12-HETE, 5-HETE, LTD_4_, and LTE_4_ (Johnson et al., 1985; Yanni et al., 1989).

15-HETE is associated with regulation of MUC5AC expression, a key determinant of mucus production in asthma (Figure 3). MUC5AC encodes Mucin 5AC, a major component of airway mucus that is constitutively produced in the human trachea and upregulated during allergic inflammation (Dhanisha et al., 2018; Adler et al., 2013). IL-13 increases *MUC5AC* mRNA expression in cultured human bronchial epithelium, which is attenuated by pharmacologic inhibition of 15-LO-1 or by siRNA targeting of *ALOX15* (Sridhar et al., 2019). Importantly, exogenous 15-HETE restores IL-13–induced MUC5AC expression, suggesting that 15-HETE mediates, at least in part, the IL-13 effect (Sridhar et al., 2019). This effect is not seen in IL-4 treated bronchial epithelium, indicating cytokine specificity (Jayawickreme et al., 1999). Furthermore, although protein kinase C activation induces *MUC5AC* expression in cultured human bronchial cells, 15-HETE reduces this effect, suggesting that 15-HETE’s modulation of *MUC5AC* expression in a context-dependent (Song et al., 2015).

**FIGURE 3:**
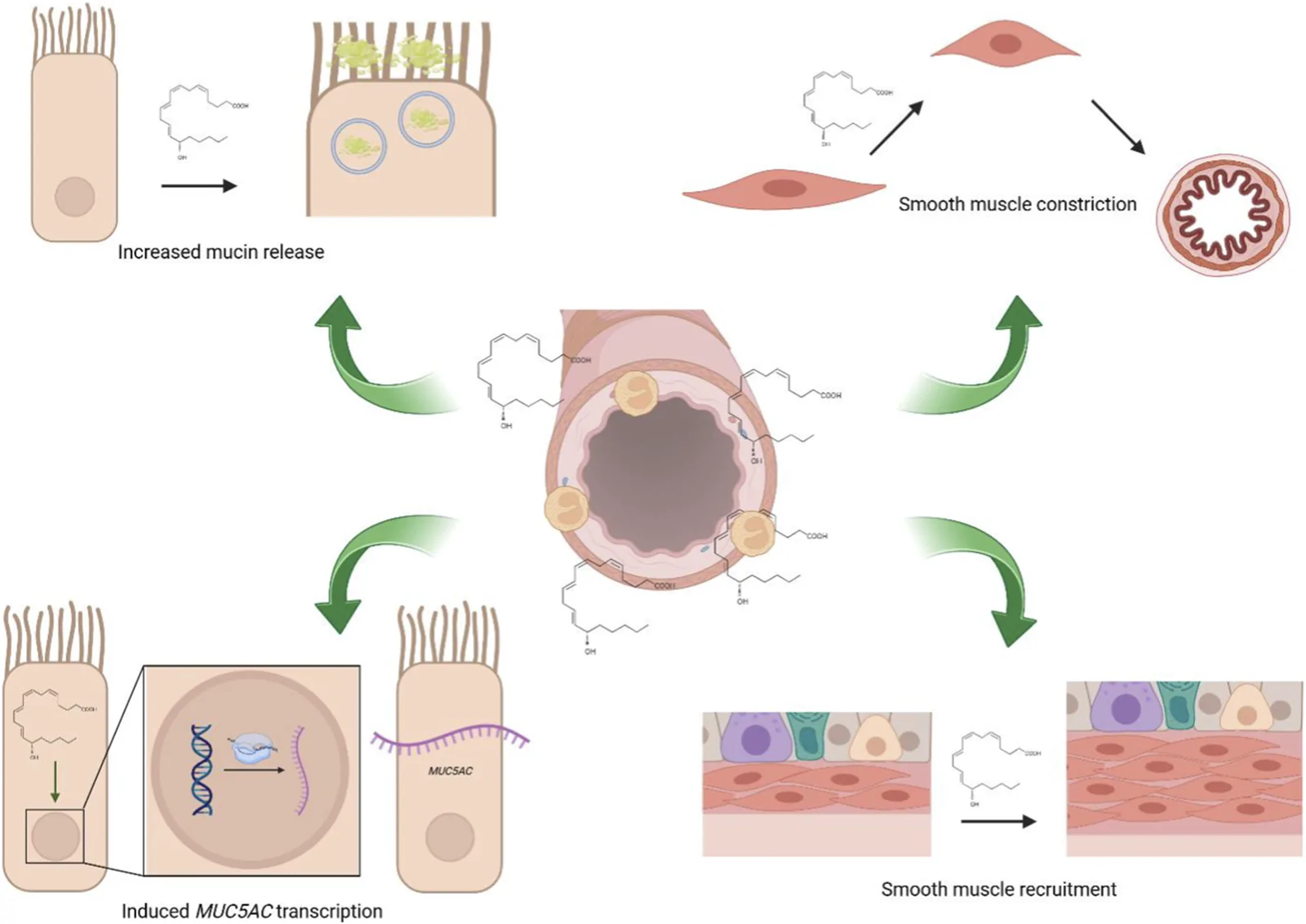
Impacts of 15-HETE on the airway. 15-HETE expressed within the airway lumen and musculature, induces several cell specific changes to create the inflamed airway seen in asthma. In the epithelium (left), 15-HETE promotes further secretion of mucin into the lumen (top left), while also inducing the expression of *MUC5AC*, increasing mucus production as well (bottom left). Within the airway smooth muscle (right), 15-HETE induces contraction of muscle cells, tightening the muscular ring surrounding the airway causing bronchoconstriction (top right), while also recruiting more muscle cells to the area, thickening the muscular layers of the airway (bottom right). Created in https://BioRender.com.

#### 3.3.3 15-HETE induced airway obstruction and remodeling

15-HETE is associated with functional airway obstruction in asthma (Alpert and Walenga, 1993). Pulmonary 15-HETE levels correlate with increased subbasement membrane thickness causing airway narrowing (Turi et al., 2023). In asthmatic patients, higher baseline 15-HETE levels are associated with reduced partial expiratory flow at 30% of vital capacity, and inhalation of 15-HETE significantly decreases FEV_1_ (Lai et al., 1990). In individuals with AERD, higher baseline 15-HETE levels predict greater improvement in FEV_1_ following aspirin desensitization suggesting a relationship between 15-HETE dynamics and airway responsiveness (Jerschow et al., 2017). Consistent with these findings, equine models of environmental allergen airway obstruction exhibit increased plasma 15-HETE levels (Gray et al., 1992). In contrast, grain dust exposure in non-asthmatic individuals decreases lung capacity and increases BALF neutrophils without altering eosinophils, lymphocytes, histamine, or 15-HETE levels, supporting a more specific association of 15-HETE with allergic eosinophilic airway inflammation (Clapp et al., 1994).

15-HETE also directly modulates airway smooth muscle function and contributes to remodeling (Figure 3). It promotes bronchial smooth muscle recruitment, proliferation, and migration (Ma et al., 2011; Chava et al., 2009). While its immediate bronchoconstrictor effect is weaker than that of histamine, 15-HETE induces a slower, but more sustained contraction of the bronchial smooth muscle (Copas et al., 1982). This may occur from 15-oxo-eicosatetraenoic acid, an oxidative derivative of 15-HETE produced by 15-hydroxyprostaglandin dehydrogenase (Kowal et al., 2017). In *ex vivo* experiments, bronchial smooth muscle contraction occurs in a dose-dependent manner 2 h after 15-HETE exposure (Salari and Schellenberg, 1991). While mediators such as 5-HETE, leukotriene C4, and carbachol induce initial contractions, 15-HETE appears to sustain prolonged contractile response, resulting in chronic airway deficits in asthma (Salari and Schellenberg, 1991). Moreover, 15-HETE acts synergistically with histamine to cause bronchial smooth muscle contraction, suggesting 15-HETE contribute to chronic asthma pathology (Copas et al., 1982).

#### 3.3.4 Inconsistent roles of 15-HETE across models and individuals

When inhaled, 15-HETE does not affect pulmonary function in normal subjects and its role in asthma is not always replicated across species (La et al., 1990). One reason is that AA metabolism differs between mice and humans: human eosinophils mainly produce 15-HETE, while murine eosinophils mainly produce 12-HETE (Archambault et al., 2022). Some asthmatics also produce more 12-HETE than 15-HETE (Kumlin et al., 1991); both are metabolites of 15-LO. Additionally, 15-HETE can be rapidly converted to 5,15-diHETE, eoxins, and lipoxins, making it difficult to detect in samples (Schwenk and Schröder, 1995). Eoxins and lipoxins serve as pro-resolving mediators in asthma. In many cell types, 15-HETE functions mainly as an intermediate, with downstream metabolites acting as eosinophil attractants (Chavis et al., 1992). Consistent with this, IgE stimulation of human bronchial cells from asthmatics does not release 15-HETE (Campbell et al., 1994), aspirin challenges in nasal polyps do not rely on 15-HETE for eosinophilic activation (Lewandowska-Polak et al., 2011), and low baseline 15-HETE individuals may still experience disease exacerbation during aspirin treatment (Kowalski et al., 2000). Similarly, lipopolysaccharide exposure does not induce 15-HETE generation in severe asthma, highlighting the context-dependent and sometimes inconsistent role of 15-HETE (Huynh et al., 2005) in asthma.

#### 3.3.5 15-HETE and eosinophilic invasion

Eosinophilic airways infiltration is a key feature of severe asthma. Eosinophils are found in airway epithelium and induced sputum of asthma patients, and in BALF of wheezing children (Sridhar et al., 2019; Al-Shaikhly et al., 2022; Krawiec et al., 2001). Symptom exacerbation in asthma often occurs in those experiencing eosinophilia (Ban et al., 2021). Eosinophils also increase in BALF as well as in whole lung sections in house dust mite challenged murine asthma models (Dai et al., 2016).

15-HETE and eosinophils are interconnected in asthma, contributing to both airway inflammation and remodeling. (Skolnik and Carnahan, 2019; Oppenheimer et al., 2022; Nadel et al., 1991). 15-HETE levels are higher in eosinophilic than in non-eosinophilic patients and eosinophils themselves express active 15-LO (Chu et al., 2002; Nadel et al., 1991). They release protein and lipid mediators that exacerbate asthmatic symptoms and their presence in the airway mirrors 15-HETE elevation in severe disease (Gleich, 2000).

15-HETE production by eosinophils occurs primarily after airway invasion. Although 15-LO is constitutively active in eosinophils, 15-HETE is produced only when eosinophils enter the lungs. Peripheral blood eosinophils do not release detectable 15-HETE, but in culture they produce 15-HETE when supplied with AA and calcium ionophore (Laviolette et al., 1986). Epithelial cells also generate 15-HETE with inflammatory stimulation. Thus, 15-HETE levels can be increased even in less severe asthma from epithelial cell production of 15-HETE (Kolmert et al., 2018). In severe eosinophilic asthma 15-HETE levels are the highest since it is produced by both eosinophils and epithelial cells (Kowalski et al., 2003; Laviolette et al., 1986).

The role of 15-HETE in eosinophil trafficking remains incompletely understood. Both *ALOX15* expression and 15-HETE concentration are positively correlated with pulmonary eosinophil count (Singh and Rao, 2019; Henderson et al., 1984). Downstream metabolites of 15-HETE such as 15-oxo-HETE and eoxins mediate chemotaxis (Marom et al., 1983; Schwenk and Schröder, 1995). Studies in 12/15-LO knockout mice show reduced airway eosinophilic density and proliferation as well as lower BALF eosinophils (Methner et al., 2026), indicating that 15-LO activity is necessary for full eosinophilic invasion (Andersson et al., 2008). At the same time, since 15-LO is responsible for generation of pro-resolving lipid mediators, including eoxins and lipoxins, 15-LO inhibition may not benefit patients with asthma (Miyata et al., 2013; Ito et al., 2025; Miyata et al., 2026). Therefore, targets downstream of 15-LO may be better options for asthma treatment.

## 4 G-protein coupled receptors (GPCRs) in asthma

GPCRs are single polypeptide chains containing an extracellular N-terminus, 7-transmembrane α-helical domains, and an intracellular C-terminus. They are the largest body of cell surface membrane proteins transducing extracellular signals to create intracellular events (Rosenbaum et al., 2009). They form targets for over one-third of all drugs used today (Lundstrom, 2009). Broadly speaking, downstream signaling acts through the G-protein subunits after GPCR activation by specific endogenous ligands or synthetic agonists.

### 4.1 GPCRs as targets for asthma treatment

In asthma, β-adrenergic receptors allow for agonists that act via Gα_s_ to increased cyclic adeno monophosphate (cAMP) to cause bronchodilation (Wendell et al., 2020; Penn et al., 2014). Similarly, Gα_q_/Gα_11_ is activated through anti-muscarinic drugs that block acetylcholine-induced bronchoconstriction (Busse et al., 2016). Drugs used to inhibit leukotrienes such as Montelukast, Zafirlukast, and Pranlukast bind to cysteinyl leukotriene receptor 1, which mostly signals through Gα_11_ to antagonize inflammation and airway hyperresponsiveness (Wu et al., 2024). Finally, chemoattractant receptor-homologous molecule expressed on Th2 cells, also known as DP2 (Prostaglandin receptor-2), is a GPCR that typically couples with Gα_i/o_ to mediate allergic inflammation (Stinson et al., 2014) and is inhibited by investigational small molecules.

### 4.2 GPR39 as target for asthma treatment

15-HETE, was recently reported as the endogenous ligand (Alkayed et al., 2022) for the orphan receptor, GPCR-39 (GPR39), part of the Ghrelin family of receptors, originally described as a Zn^++^ receptor because it was activated by Zn^++^ in supraphysiological concentrations. Later, it was proposed as the receptor for Obestatin that was subsequently refuted (Holst et al., 2004; Popovics and Stewart, 2011). More recently it was found to be present in vascular smooth muscle cells (VSMCs) in the mouse heart where it responded to physiological concentrations of 15-HETE, the endogenous agonist, and 14,15-eicosatrienoic acid (14,15-EET), the endogenous antagonist (Alkayed et al., 2022). Figure 4 illustrates downstream signaling events after activation of GPR39.

**FIGURE 4:**
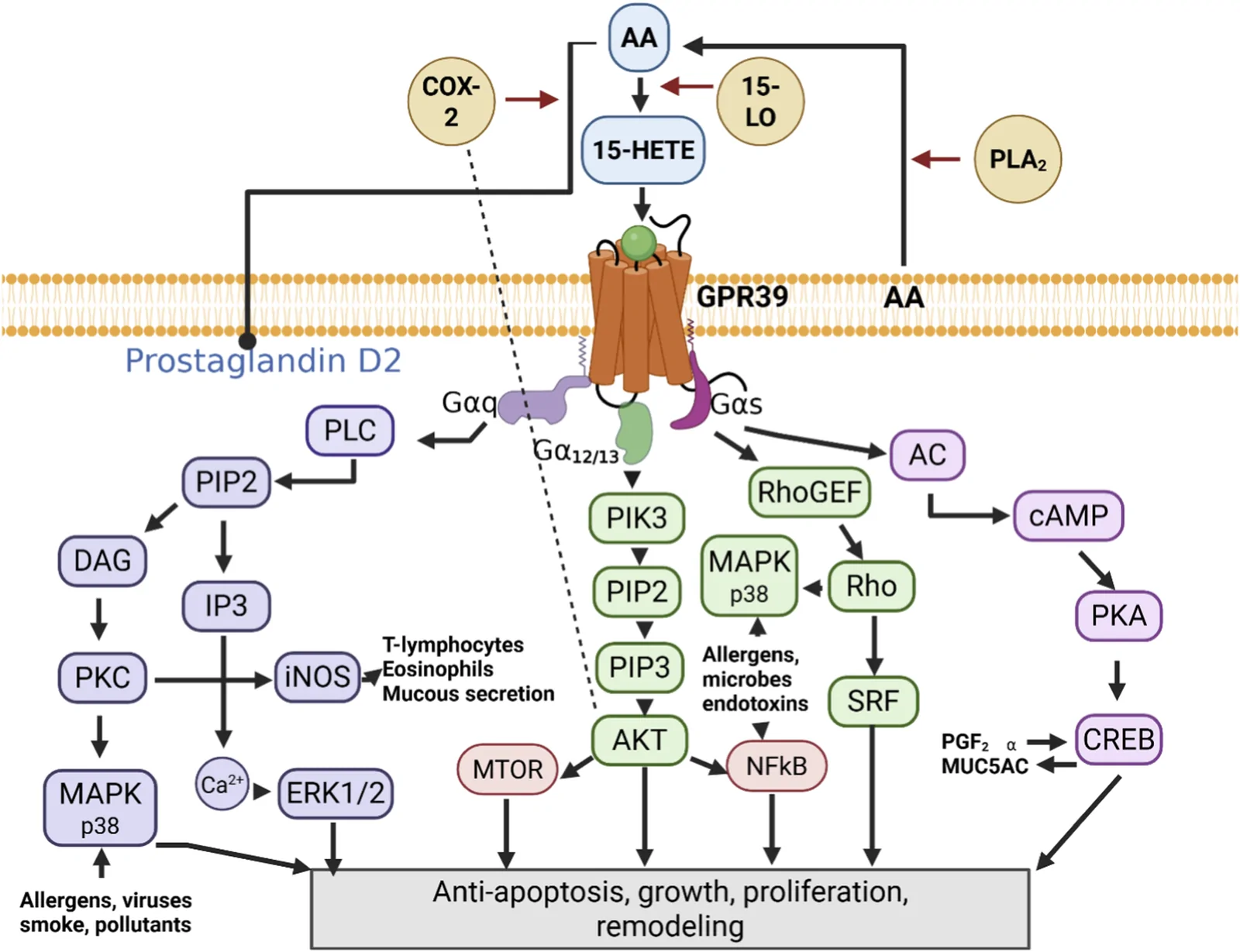
Signaling upstream and downstream of GPR39. Upstream of GPR39 is PLA_2_, which releases AA from plasma membrane. AA is then converted by 15-LO to 15-HETE, which then act as a GPR39 agonist and activate several G-proteins (Gα_q_, Gα_s_, and Gα_12/13_), initiating downstream signaling. Gα_q_ activates protein kinase C, increasing levels of iNOS. iNOS is also activated by T-lymphocytes, eosinophils, and mucous secretion. Gα_12/13_ stimulation results in activation of the PI3K-AKT pathway that influences expressions of MTOR and NFκB. Both Gαq and Gα12/13 pathways stimulate the rho pathway with activation of ROCK and MAPK. Both MAPK and NFκB are activated by allergens, microbes and endotoxins. Gα_s_ stimulation results in increased CREB, which is also activated by PGF2α and MUC5AC. All three pathways ultimately lead to cell proliferation and growth through transcription factors within the nucleus. 15-HETE = 15-hydroxyeicosatetraenoic acid; 15-LOX = 15-lipoxygenase; cAMP = cyclic adenosine monophosphate; CREB = cAMP response element binding protein; COX2 = cyclooxygenase 2; DAG = 1,2 diacylglycerol; eNOS = endothelial nitric oxide; ERK = extracellular signal related kinase; iNOS = inducible nitric oxide; IP3 = inositol 1,4,5 triphosphate; MAPK = mitogen-activated protein kinase; MTOR = mechanistic target for rafamycin; NFκB = nuclear factor κB; PGE = prostaglandin E; PGF2 = prostaglandin F2; PIP2 = phosphatidylinositol 4,5 biphosphate; PIP3 = phosphatidylinositol (Prevention, 2024; Levy et al., 2023; World Health Organization, 2026)-trisphosphate; PLA_2_ = phospholipase A_2;_ ROCK = Rho-associated coiled-coil kinase. Created in https://BioRender.com.

GPR39 has significant constitutive activity via its G_αq_ and G_α12/13_ subunits (Holst et al., 2004; Popovics and Stewart, 2011). When stimulated by high doses of Zn^++^ or synthetic agonists, in addition to further activation of Gα_q_ and Gα_12/13,_ Gα_s_ is also activated (Holst et al., 2004; Popovics and Stewart, 2011). Gα_q_ activation by 15-HETE increases cytosolic Ca^++^ and causes extracellular signal related kinase (ERK) phosphorylation (Alkayed et al., 2022). Increased cytosolic Ca^++^ causes VSMC contraction (Alkayed et al., 2022), while phosphorylated ERK translocates to the nucleus and activates several transcription factors (Busse et al., 2016; Wu et al., 2024). ERK is activated in asthma (Hao and Garcia-de-Alba-Rivas, 2023).

Activation of Gα_q_ increases inducible nitric oxide (iNOS) through stimulation of protein kinase C, which is overexpressed in asthmatic lungs (Prado et al., 2011; Tufvesson et al., 2011). iNOS is significantly upregulated in the airway epithelial cells and macrophages of patients with asthma, primarily driven by T2-high inflammation (IL-13). It acts as a major source of excess nitric oxide (NO) in the airways, contributing to eosinophilic inflammation, mucus secretion, vascular permeability, and remodeling (Holst et al., 2004). Fractional exhaled NO provides prognostic information in severe asthma (Tufvesson et al., 2011).

Activated Gα_12/13_ stimulates the AKT-phosphatidylinositol 3-kinase (PI3K) pathway, which affects several nuclear transcriptional factors leading to cell growth and proliferation as well as reduced apoptosis in asthma (Uehara et al., 2025). It acts as an attractant of eosinophils (Song et al., 2025) and participates in inflammation (Yoo et al., 2017). The AKT-PI3K pathway also acts as a regulator of COX2, which is responsible for production of prostaglandins, some of which are detrimental in asthma (Claar et al., 2015). It further regulates the redox-sensitive transcription factor, nuclear factor (NF)-κB, an important player in inflammatory networks that regulate cytokine activity in airway pathology (Schuliga, 2015). Glucocorticoids inhibit NF-κB gene transcription. Finally, The AKT-PI3K pathway also activates mechanistic target of rapamycin (MTOR) that regulates metaplasia of mucous producing airway cells (Kudrna et al., 2025).

Activation of both Gα_q_ and Gα_12/13_ stimulates the Rho pathway leading to cell proliferation and growth through, among other molecules, Rho-associated coiled-coil kinase (ROCK). This pathway is activated in asthma leading to bronchial smooth muscle hyper-responsiveness (Zhang et al., 2020). Small molecules have been developed to block this pathway (Gondas et al., 2024). Finally, Gα_s_ activation stimulates cAMP response element binding protein (CREB), a kinase inducible transcription factor that contributes to increased proliferation and migration of airway smooth muscle cells, driving airway remodeling (Chung et al., 2009). It also promotes a Th2 response, increasing pro-inflammatory factors (Harker and Lloyd, 2023). Phosphorylated CREB expression is increased in untreated and steroid-dependent asthmatics compared to healthy controls (Harker and Lloyd, 2023). Based on these findings, it appears reasonable to suggest GPR39 as a therapeutic target in eosinophilic asthma. It has been shown that genetic deletion of GPR39 inhibits the Gα_q_, Gα_s_, and Gα_12/13_ pathways in the lung (Methner et al., 2026).

### 4.3 Clinical relevance and therapeutic implications

Currently asthma therapies primarily address symptoms. Inhaled corticosteroids, long-acting β2-agonists, and tiotropium reduce inflammation and bronchospasm in mild or moderate asthma but often fail in severe asthma (Levy et al., 2023; World Health Organization, 2026). Consequently, research has focused on novel strategies that intervene at different stages of AA metabolism and downstream signaling (Figure 5). Inhibiting AA release through reduction in PLA_2_ activity could theoretically reduce eicosanoid, prostaglandin, and leukotriene production; however, the broad-spectrum PLA_2_ inhibitor LY333013 did not significantly alter allergen-induced bronchoconstriction in humans (Bowton et al., 2005). Similarly, 15-HETE production could be reduced by inhibiting 15-LO, but several lipoxygenase inhibitors in development and preclinical trials (Chung et al., 2009), including 15-LO inhibitors (Bowton et al., 2005), have been tried in asthma with limited success probably because 15-LO is also required for asthma alleviation through generation of pre-resolving mediators.

**FIGURE 5:**
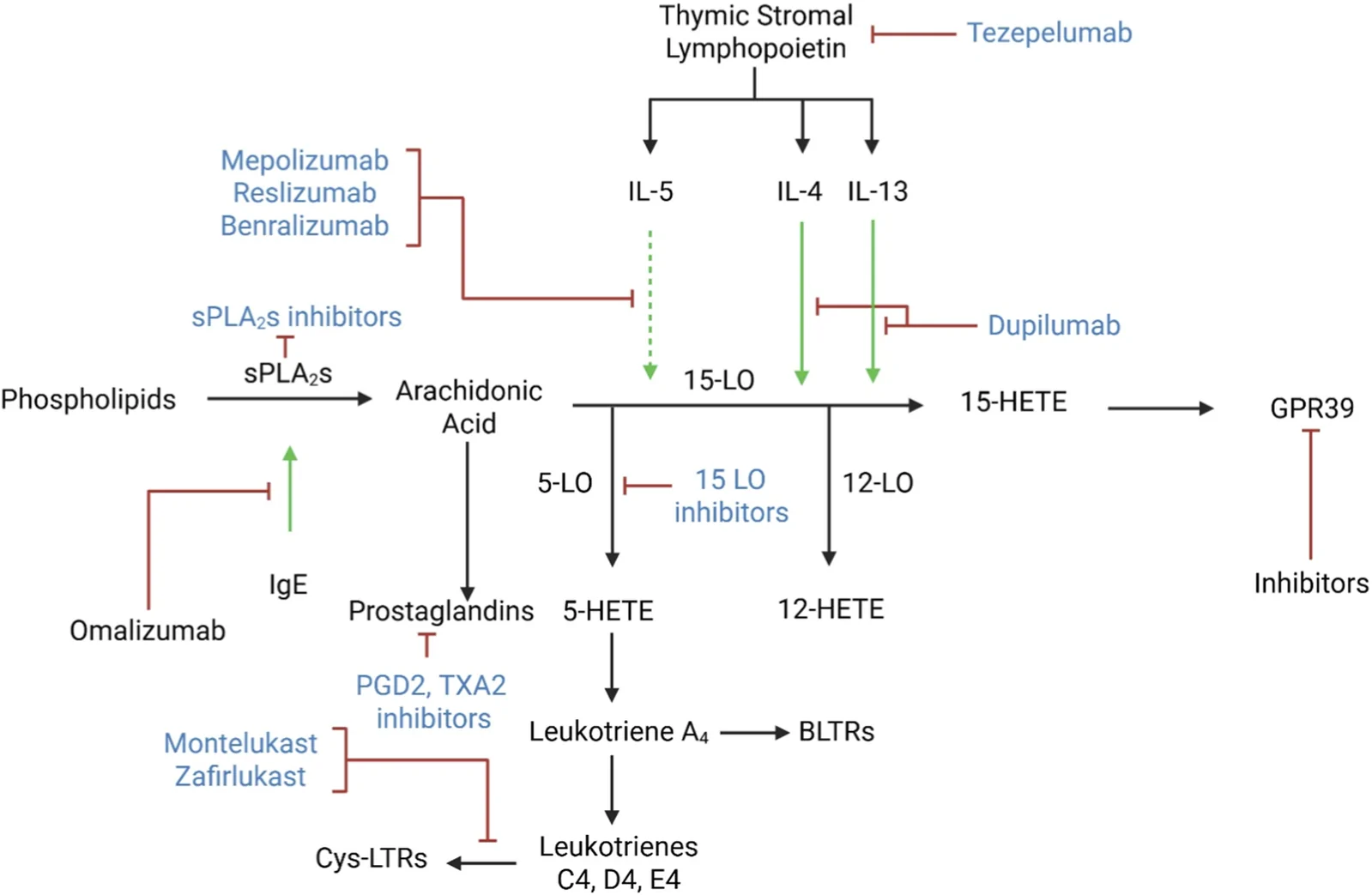
Drug targets for asthma. Current and future targets are AA metabolites and their receptors as well as cytokines and their receptors. Drugs classes as well as names are in blue. BLTRs = leukotriene B receptors. Cys-LTR = Cysteinyl Leukotriene Receptor. Created in https://BioRender.com.

Targeting specific downstream mediators of AA metabolism has had mixed results. Prostaglandin D2 and Thromboxane A2 contribute to inflammation, hypersecretion, and bronchospasm. While receptor antagonists or enzyme inhibitors for these mediators show some efficacy in mild to moderate asthma, they have not matched the success of newer targeted therapies (Poff and Balazy, 2004). Leukotriene receptor antagonists, such as montelukast and zafirlukast, improve symptoms and reduce exacerbations, particularly when combined with low-dose inhaled steroids, but they do not fully restore pulmonary function in uncontrolled asthma (Kerstjens and Gosens, 2020; Dessanges et al., 1999; Edelman et al., 2000). Consequently, recent discovery of GPR39 as a receptor for 15-HETE opens a new avenue for targeted therapy.

## 5 Conclusion/future directions

The AA–15-HETE axis represents a critical link between lipid metabolism and immune regulation in eosinophilic asthma. AA is metabolized through enzymatic pathways, including 15-LO and cyclooxygenases, to generate 15-HETE, a bioactive lipid mediator, tissue levels of which are elevated in asthma and associated with disease severity. 15-HETE contributes to key features of eosinophilic asthma, including eosinophil recruitment, mucus hypersecretion, and airway remodeling (central illustration, Figure 6). Despite these observations, the downstream signaling mechanisms through which 15-HETE exerts its effects remain incompletely understood.

**FIGURE 6:**
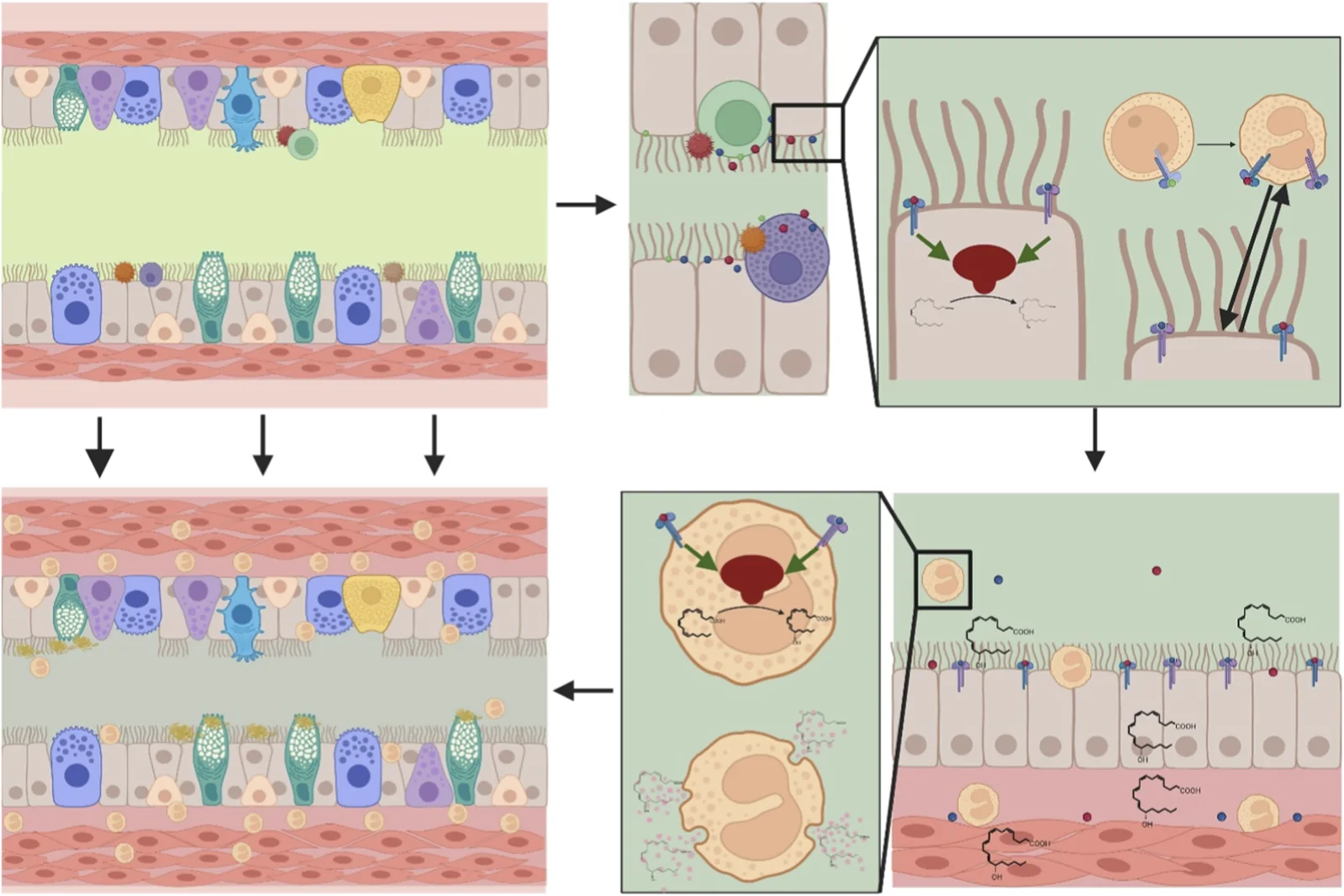
Central Illustration. Cytokines and 15-LO in eosinophilic asthma. Allergens enter the healthy airway (top left) triggering response from mast cells and CD4^+^ lymphocytes along the epithelium (top middle). Release of IL-4 and IL-13 activates 15-LO, which converts AA into 15-HETE (top right), as well as induce eosinophilic migration. IL-5 induces maturation and sequestration of eosinophiles into the airway (top right). 15 HETE penetrates the lumen, epithelium, subbasement membrane, and smooth musculature (bottom right). Eosinophils, attracted to the airway by IL-4 and IL-13, further convert AA acid to 15-HETE. Signaling from IL-4, IL-13, and eosinophils release additional 15-HETE. This results in inflammation, smooth muscle proliferation, bronchoconstriction, basement membrane thickening, and mucus secretion, resulting in the eosinophilic asthma phenotype (bottom left). Created in https://BioRender.com.

GPR39 has recently been proposed as a potential receptor for 15-HETE, linking lipid metabolism to intracellular signaling pathways involved in inflammation and tissue remodeling. Although the role of GPR39 in asthma has not been studied, its ability to mediate responses to bioactive lipids suggests it may serve as a critical signaling node in eosinophilic inflammation. Taken together, these findings support a model in which the AA–15-HETE–GPR39 axis contributes to the pathogenesis of eosinophilic asthma. Targeting the 15-HETE–GPR39 axis may therefore represent a novel and underexplored therapeutic strategy for modulating airway inflammation and remodeling.

## References

[B1] Adler K. B. Tuvim M. J. Dickey B. F. (2013). Regulated mucin secretion from airway epithelial cells. Front. Endocrinol. (Lausanne) 4, 129. 10.3389/fendo.2013.00129 24065956 PMC3776272

[B2] Al-Shaikhly T. Murphy R. C. Parker A. Lai Y. Altman M. C. Larmore M. (2022). Location of eosinophils in the airway wall is critical for specific features of airway hyperresponsiveness and T2 inflammation in asthma. Eur. Respir. J. 60, 2101865. 10.1183/13993003.01865-2021 PMC9704864 35027395

[B3] Alkayed N. J. Cao Z. Qian Z. Y. Nagarajan S. Liu X. Nelson J. W. (2022). Control of coronary vascular resistance by eicosanoids via a novel GPCR. Am. J. Physiol. 322, C1011–C1021. 10.1152/ajpcell.00454.2021 35385329 PMC9255704

[B4] Alpert S. E. Walenga R. W. (1993). Human tracheal epithelial cells selectively incorporate 15-hydroxyeicosatetraenoic acid into phosphatidylinositol. Am. J. Respir. Cell Mol. Biol. 8, 273–281. 10.1165/ajrcmb/8.3.273 8448017

[B5] Alpert S. E. Walenga R. W. (1995). Ozone exposure of human tracheal epithelial cells inactivates cyclooxygenase and increases 15-HETE production. Am. J. Physiol. 269, L734–L743. 10.1152/ajplung.1995.269.6.L734 8572235

[B6] Andersson C. K. Claesson H. E. Rydell-Tormanen K. Swedmark S. Hallgren A. Erjefalt J. S. (2008). Mice lacking 12/15-lipoxygenase have attenuated airway allergic inflammation and remodeling. Am. J. Respir. Cell. Mol. Biol. 39, 648–656. 10.1165/rcmb.2007-0443OC 18511709

[B7] Archambault A. S. Brassard J. Bernatchez E. Martin C. Di Marzo V. Laviolette M. (2022). Human and mouse eosinophils differ in their ability to biosynthesize eicosanoids, docosanoids, the endocannabinoid 2-Arachidonoyl-glycerol and its congeners. Cells 11, 14. 10.3390/cells11010141 PMC8750928 35011703

[B8] Ban G. Y. Kim S. H. Park H. S. (2021). Persistent eosinophilic inflammation in adult asthmatics with high serum and urine levels of leukotriene E(4). J. Asthma Allergy 14, 1219–1230. 10.2147/jaa.s325499 PMC8520485 34675552

[B9] Bowton D. L. Seeds M. C. Fasano M. B. Goldsmith B. Bass D. A. (1997). Phospholipase A2 and arachidonate increase in bronchoalveolar lavage fluid after inhaled antigen challenge in asthmatics. Am. J. Respir. Crit. Care. Med. 155, 421–425. 10.1164/ajrccm.155.2.9032172 9032172

[B10] Bowton D. L. Dmitrienko A. A. A. Israel E. Zeiher B. G. Sides G. D. (2005). England: Informa UK ltd. Impact of a soluble phospholipase A2 inhibitor on inhaled allergen challenge in subjects with asthma *J. Asthma* . J. Asthma 42, 65–71. 10.1081/jas-200044748 15801331

[B11] Brash A. R. Boeglin W. E. Chang M. S. (1997). Discovery of a second 15S-lipoxygenase in humans. Proc. Natl. Acad. Sci. U.S.A. 94, 6148–6152. 10.1073/pnas.94.12.6148 9177185 PMC21017

[B12] Brown C. D. Kilty I. Yeadon M. Jenkinson S. (2001). Regulation of 15-lipoxygenase isozymes and mucin secretion by cytokines in cultured normal human bronchial epithelial cells. Inflamm. Res. 50, 321–326. 10.1007/PL00000251 11475334

[B13] Brunnstrom A. Tryselius Y. Feltenmark S. Andersson E. Leksell H. James A. (2015). On the biosynthesis of 15-HETE and eoxin C4 by human airway epithelial cells. Prostagl. Other Lipid Mediat 121, 83–90. 10.1016/j.prostaglandins.2015.04.010 26026713

[B14] Busse W. W. Dahl R. Jenkins C. Cruz A. A. (2016). Long-acting muscarinic antagonists: a potential add-on therapy in the treatment of asthma? Eur. Respir. Rev. 139, 54–64. 10.1183/16000617.0052-2015 26929422 PMC9487660

[B15] Campbell A. M. Vignola A. M. Chanez P. Godard P. Bousquet J. (1994). Low-affinity receptor for IgE on human bronchial epithelial cells in asthma. Immunology 82, 506–508. 7835911 PMC1414915

[B16] Carr T. F. Berdnikovs S. Simon H. U. Bochner B. S. Rosenwasser L. J. (2016). Eosinophilic bioactivities in severe asthma. World Allergy Organ J. 9, 21. 10.1186/s40413-016-0112-5 27386041 PMC4924237

[B17] Chang Y. S. Kim Y. K. Bahn J. W. Kim S. H. Park H. W. Kim T. B. (2005). Comparison of asthma phenotypes using different sensitizing protocols in mice. Korean J. Intern. Med. 20, 152–158. 10.3904/kjim.2005.20.2.152 16134771 PMC3891385

[B18] Chava K. R. Karpurapu M. Wang D. Bhanoori M. Kundumani-Sridharan V. Zhang Q. (2009). CREB-mediated IL-6 expression is required for 15(S)-hydroxy-eicosatetraenoic acid-induced vascular smooth muscle cell migration. Arterioscler. Thromb. Vasc. Biol. 29, 809–815. 10.1161/ATVBAHA.109.185777 19342597 PMC2724759

[B19] Chavis C. Godard P. Crastes de Paulet A. Damon M. (1992). Formation of lipoxins and leukotrienes by human alveolar macrophages incubated with 15(S)-HETE: a model for cellular cooperation between macrophages and airway epithelial cells. Eicosanoids 5, 203–211. 1337977

[B20] Chen L. C. Tseng H. M. Kuo M. L. Chiu C. Y. Liao S. L. Su K. W. (2021). Levels of 15-HETE and TXB(2) in exhaled breath condensates as markers for diagnosis of childhood asthma and its therapeutic outcome. Pediatr. Allergy Immunol. 32, 1673–1680. 10.1111/pai.13587 34176158

[B21] Chu H. W. Balzar S. Westcott J. Y. Trudeau J. B. Sun Y. Conrad D. J. (2002). Expression and activation of 15-lipoxygenase pathway in severe asthma: relationship to eosinophilic phenotype and collagen deposition. Clin. Exp. Allergy. 32, 1558–1565. 10.1046/j.1365-2222.2002.01477.x 12569975

[B22] Chung W. C. Ryu S. H. Sun H. Zeldin D. C. Koo J. S. (2009). CREB mediates prostaglandin F2alpha-induced MUC5AC overexpression. J. Immunol. 182, 2349–2356. 10.4049/jimmunol.0713637 19201889 PMC2756474

[B23] Claar D. Hartert T. V. Peebles R. S. Jr (2015). The role of prostaglandins in allergic lung inflammation and asthma. Expert Rev. Respir. Med. 9, 55–72. 10.1586/17476348.2015.992783 25541289 PMC4380345

[B24] Clapp W. D. Becker S. Quay J. Watt J. L. Thorne P. S. Frees K. L. (1994). Grain dust-induced airflow obstruction and inflammation of the lower respiratory tract. Am. J. Respir. Crit. Care Med. 150, 611–617. 10.1164/ajrccm.150.3.8087327 8087327

[B25] Copas J. L. Borgeat P. Gardiner P. J. (1982). The actions of 5-12-and 15-HETE on tracheobronchial smooth muscle. Prostagl. Leukot. Med. 8, 105–114. 10.1016/s0262-1746(82)80002-4 6952280

[B26] Corren J. Du E. Gubbi A. Vanlandingham R. (2021). Variability in blood eosinophil counts in patients with eosinophilic asthma. J. Allergy Clin. Immunol. Pract. 9, 1224–1231. 10.1016/j.jaip.2020.10.033 33130147

[B27] Dai C. Yao X. Gordon E. M. Barochia A. Cuento R. A. Kaler M. (2016). A CCL24-dependent pathway augments eosinophilic airway inflammation in house dust mite-challenged Cd163−/− mice. Mucosal. Immunol. 9, 702–717. 10.1038/mi.2015.94 26376364 PMC4794428

[B28] de Groot J. C. Ten Brinke A. Bel E. H. (2015). Management of the patient with eosinophilic asthma: a new era begins. ERJ. Open. Res. 1, 00024–02015. 10.1183/23120541.00024-2015 27730141 PMC5005141

[B29] Denton E. Price D. B. Tran T. N. Canonica G. W. Menzies-Gow A. FitzGerald J. M. (2021). Cluster analysis of inflammatory biomarker expression in the international severe asthma registry. J. Allergy Clin. Immunol. Pract. 9, 2680–2688.e7. 10.1016/j.jaip.2021.02.059 33744476

[B30] Dessanges J. F. Préfaut C. Taytard A. Matran R. Naya I. Compagnon A. (1999). The effect of zafirlukast on repetitive exercise-induced bronchoconstriction: the possible role of leukotrienes in exercise-induced refractoriness. J. Allergy. Clin. Immunol. 104, 1155–1161. 10.1016/s0091-6749(99)70007-2 10588995

[B31] Dhanisha S. S. Guruvayoorappan C. Drishya S. Abeesh P. (2018). Mucins: structural diversity, biosynthesis, its role in pathogenesis and as possible therapeutic targets. Crit. Rev. Oncol. Hematol. 122, 98–122. 10.1016/j.critrevonc.2017.12.006 29458795

[B32] Edelman J. M. Turpin J. A. Bronsky E. A. Grossman J. Kemp J. P. Ghannam A. F. (2000). Oral montelukast compared with inhaled salmeterol to prevent exercise-induced bronchoconstriction. A randomized, double-blind trial. Exercise study group. Ann. Intern .Med. 132, 97–104. 10.7326/0003-4819-132-2-200001180-00002 10644288

[B33] Endo Y. Hirahara K. Yagi R. Tumes D. J. Nakayama T. (2014). Pathogenic memory type Th2 cells in allergic inflammation. Trends Immunol. 35, 69–78. 10.1016/j.it.2013.11.003 24332592

[B34] Gan Q. F. Sigal E. Browner M. F. (1997). A model of arachidonic acid binding for 15-lipoxygenase. Adv. Exp. Med. Biol. 433, 435–438. 10.1007/978-1-4899-1810-9_96 9561189

[B35] Gleich G. J. (2000). Mechanisms of eosinophil-associated inflammation. J. Allergy. Clin. Immunol. 105, 651–663. 10.1067/mai.2000.105712 10756213

[B36] Gondas E. Mazerik J. Dohal M. Balentova S. Pokusa M. Vargova D. (2024). Anti-astmatic effect of rock inhibitor, gsk429286 a, in experimentally induced allergic airway inflammation. Int. J. Immunopathol. Pharmacol. 38, 3946320241282949. 10.1177/03946320241282949 PMC11418236 39305209

[B37] Gray P. R. Derksen F. J. Broadstone R. V. Robinson N. E. Johnson H. G. Olson N. C. (1992). Increased pulmonary production of immunoreactive 15-hydroxyeicosatetraenoic acid in an animal model of asthma. Am. Rev. Respir. Dis. 145, 1092–1097. 10.1164/ajrccm/145.5.1092 1586052

[B38] Gulliksson M. Brunnstrom A. Johannesson M. Backman L. Nilsson G. Harvima I. (2007). Expression of 15-lipoxygenase type-1 in human mast cells. Biochim. Biophys. Acta. 1771, 1156–1165. 10.1016/j.bbalip.2007.06.001 17662651

[B39] Hajek A. R. Lindley A. R. Favoreto S. Jr. Carter R. Schleimer R. P. Kuperman D. A. (2008). 12/15-Lipoxygenase deficiency protects mice from allergic airways inflammation and increases secretory IgA levels. J. Allergy Clin. Immunol. 122, 633–639. 10.1016/j.jaci.2008.06.021 18692885 PMC2802267

[B40] Hallstrand T. S. Lai Y. Altemeier W. A. Appel C. L. Johnson B. Frevert C. W. (2013). Regulation and function of epithelial secreted phospholipase A2 group X in asthma. Am. J. Respir. Crit. Care Med. 188, 42–50. 10.1164/rccm.201301-0084OC 23614662 PMC3735246

[B41] Hamu-Tanoue A. Takagi K. Taketomi Y. Miki Y. Nishito Y. Kano K. (2024). Group III secreted phospholipase A(2) -driven lysophospholipid pathway protects against allergic asthma. FASEB J. 38, e23428. 10.1096/fj.202301976r 38236184

[B42] Hao Y. Garcia-de-Alba-Rivas C. (2023). Selective inhibition of extracellular signal-regulated kinases 1 and 2: when less is more. Am. J. Respir. Cell. Mol. Biol. 68, 1–2. 10.1165/rcmb.2022-0376ed PMC9817910 36227721

[B43] Harker J. A. Lloyd C. M. (2023). T helper 2 cells in asthma. J. Exp. Med. 220, e20221094. 10.1084/jem.20221094 37163370 PMC10174188

[B44] Henderson W. R. Harley J. B. Fauci A. S. (1984). Arachidonic acid metabolism in normal and hypereosinophilic syndrome human eosinophils: generation of leukotrienes B4, C4, D4 and 15-lipoxygenase products. Immunology 51, 679–686. 6323307 PMC1454541

[B45] Hill E. M. Eling T. Nettesheim P. (1998). Changes in expression of 15-lipoxygenase and prostaglandin-H synthase during differentiation of human tracheobronchial epithelial cells. Am. J. Respir. Cell. Mol. Biol. 18, 662–669. 10.1165/ajrcmb.18.5.2985 9569236

[B46] Holst B. Holliday N. D. Bach A. Elling C. E. Cox H. M. Schwartz T. W. (2004). Common structural basis for constitutive activity of the ghrelin receptor family. J. Biol. Chem. 279, 53806–53817. 10.1074/jbc.M407676200 15383539

[B47] Holtzman M. J. (1992). Arachidonic acid metabolism in airway epithelial cells. Ann. Rev. Physiol. 54, 303–329. 10.1146/annurev.ph.54.030192.001511 1562177

[B48] Hussain M. Liu G. (2024). Eosinophilic asthma: pathophysiology and therapeutic Horizons. Cells 13, 384. 10.3390/cells13050384 PMC10931088 38474348

[B49] Huynh M. L. Malcolm K. C. Kotaru C. Tilstra J. A. Westcott J. Y. Fadok V. A. (2005). Defective apoptotic cell phagocytosis attenuates prostaglandin E2 and 15-hydroxyeicosatetraenoic acid in severe asthma alveolar macrophages. Am. J. Respir. Crit. Care Med. 172, 972–979. 10.1164/rccm.200501-035OC 16040786

[B50] Ito E. Hayashizaki R. Hosaka T. Yamane T. Miyata J. Isobe Y. (2025). Eosinophils and pleural macrophages counter regulate il-33-elicited airway inflammation via the 12/15-lipoxygenase pathway. Front. Immunol. 16, 1565670. 10.3389/fimmu.2025.1565670 PMC12043891 40313934

[B51] Ivanov I. Kuhn H. Heydeck D. (2015). Structural and functional biology of arachidonic acid 15-lipoxygenase-1 (ALOX15). Gene 573, 1–32. 10.1016/j.gene.2015.07.073 26216303 PMC6728142

[B52] James A. Daham K. Backman L. Brunnstrom A. Tingvall T. Kumlin M. (2013). The influence of aspirin on release of eoxin C4, leukotriene C4 and 15-HETE, in eosinophilic granulocytes isolated from patients with asthma. Int. Arch. Allergy Immunol. 162, 135–142. 10.1159/000351422 23921438

[B53] Jayawickreme S. P. Gray T. Nettesheim P. Eling T. (1999). Regulation of 15-lipoxygenase expression and mucus secretion by IL-4 in human bronchial epithelial cells. Am. J. Physiol. 276, L596–L603. 10.1152/ajplung.1999.276.4.L596 10198357

[B54] Jedrzejczak-Czechowicz M. Lewandowska-Polak A. Bienkiewicz B. Kowalski M. L. (2008). Involvement of 15-lipoxygenase and prostaglandin EP receptors in aspirin-triggered 15-hydroxyeicosatetraenoic acid generation in aspirin-sensitive asthmatics. Clin. Exp. Allergy. 38, 1108–1116. 10.1111/j.1365-2222.2008.02989.x 18462455

[B55] Jerschow E. Edin M. L. Pelletier T. Abuzeid W. M. Akbar N. A. Gibber M. (2017). Plasma 15-Hydroxyeicosatetraenoic acid predicts treatment outcomes in aspirin-exacerbated respiratory disease. J. Allergy Clin. Immunol. Pract. 5, 998–1007. 10.1016/j.jaip.2016.11.021 28159558 PMC5491381

[B56] Johnson H. G. McNee M. L. Sun F. F. (1985). 15-Hydroxyeicosatetraenoic acid is a potent inflammatory mediator and agonist of canine tracheal mucus secretion. Am. Rev. Respir. Dis. 131, 917–922. 10.1164/arrd.1985.131.6.917 3923884

[B57] Kerstjens H. Gosens R. (2020). Prostaglandin D_2_: the end of a story or just the beginning? Lancet Respir. Med. 9, 2–3. 10.1016/S2213-2600(20)30449-5 33160458

[B58] Kolmert J. Pineiro-Hermida S. Hamberg M. Gregory J. A. Lopez I. P. Fauland A. (2018). Prominent release of lipoxygenase generated mediators in a murine house dust mite-induced asthma model. Prostagl. Other Lipid Mediat 137, 20–29. 10.1016/j.prostaglandins.2018.05.005 29763661

[B59] Kowal K. Gielicz A. Sanak M. (2017). The effect of allergen-induced bronchoconstriction on concentration of 5-oxo-ETE in exhaled breath condensate of house dust mite-allergic patients. Clin. Exp. Allergy. 47, 1253–1262. 10.1111/cea.12990 28763131

[B60] Kowalski M. L. Pawliczak R. Wozniak J. Siuda K. Poniatowska M. Iwaszkiewicz J. (2000). Differential metabolism of arachidonic acid in nasal polyp epithelial cells cultured from aspirin-sensitive and aspirin-tolerant patients. Am. J. Respir. Crit. Care Med. 161, 391–398. 10.1164/ajrccm.161.2.9902034 10673176

[B61] Kowalski M. L. Ptasinska A. Bienkiewicz B. Pawliczak R. DuBuske L. (2003). Differential effects of aspirin and misoprostol on 15-hydroxyeicosatetraenoic acid generation by leukocytes from aspirin-sensitive asthmatic patients. J. Allergy Clin. Immunol. 112, 505–512. 10.1016/s0091-6749(03)01716-0 13679808

[B62] Kowalski M. L. Ptasinska A. Jedrzejczak M. Bienkiewicz B. Cieslak M. Grzegorczyk J. (2005). Aspirin-triggered 15-HETE generation in peripheral blood leukocytes is a specific and sensitive aspirin-sensitive patients identification test (ASPITest). Allergy 60, 1139–1145. 10.1111/j.1398-9995.2005.00836.x 16076298

[B63] Kowalski M. L. Borowiec M. Kurowski M. Pawliczak R. (2007). Alternative splicing of cyclooxygenase-1 gene: altered expression in leucocytes from patients with bronchial asthma and association with aspirin-induced 15-HETE release. Allergy 62, 628–634. 10.1111/j.1398-9995.2007.01366.x 17508966

[B64] Krawiec M. E. Westcott J. Y. Chu H. W. Balzar S. Trudeau J. B. Schwartz L. B. (2001). Persistent wheezing in very young children is associated with lower respiratory inflammation. Am. J. Respir. Crit. Care Med. 163, 1338–1343. 10.1164/ajrccm.163.6.2005116 11371398

[B65] Kudrna K. M. Vilches L. F. Eilers E. M. Maurya S. K. Brody S. L. Horani A. (2025). MTOR signaling regulates the development of airway mucous cell metaplasia associated with severe asthma. JCI Insight 10, e187904. 10.1172/jci.insight.187904 40446022 PMC12288895

[B66] Kuhn H. Belkner J. Wiesner R. Brash A. R. (1990). Oxygenation of biological membranes by the pure reticulocyte lipoxygenase. J. Biol. Chem. 265, 18351–18361. 2120232

[B67] Kumlin M. Ohlson E. Bjorck T. Hamberg M. Granstrom E. Dahlen B. (1991). 15(S)-hydroxyeicosatetraenoic acid (15-HETE) is the major arachidonic acid metabolite in human bronchi. Adv. Prostaglandin. Thromboxane Leukot. Res. 21A, 441–444. 1825578

[B68] Kytikova O. Y. Kovalenko I. S. Novgorodtseva T. P. Denisenko Y. K. (2024). The role of hydroxyeicosatetraenoic acids in the regulation of inflammation in bronchial asthma. Dokl. Biochem. Biophys. 519, 512–520. 10.1134/s1607672924701126 39283556

[B69] Lai C. K. Phillips G. D. Jenkins J. R. Holgate S. T. (1990). The effect of inhaled 15-(s)-hydroxyeicosatetraenoic acid (15-HETE) on airway calibre and non-specific responsiveness in normal and asthmatic human subjects. Eur. Respir. J. 3, 38–45. 2311730

[B70] Lai C. K. Polosa R. Holgate S. T. (1990). Effect of 15-(s)-hydroxyeicosatetraenoic acid on allergen-induced asthmatic responses. Am. Rev. Respir. Dis. 141, 1423–1427. 10.1164/ajrccm/141.6.1423 2190507

[B71] Laviolette M. Picard S. Braquet P. Borgeat P. (1986). Comparison of 5- and 15-lipoxygenase activities in blood and alveolar leukocyte preparations from normal subjects and patients with eosinophilia. Prostagl. Leukot. Med. 23, 191–199. 10.1016/0262-1746(86)90185-x 3020586

[B72] Levy M. L. Bacharier L. B. Bateman E. Boulet L. P. Brightling C. Buhl R. (2023). Key recommendations for primary care from the 2022 global initiative for asthma (GINA) update. NPJ. Prim. Care Respir. Med. 33, 7. 10.1038/s41533-023-00330-1 PMC9907191 36754956

[B73] Lewandowska-Polak A. Jedrzejczak-Czechowicz M. Makowska J. S. Jarzebska M. Jankowski A. Kowalski M. L. (2011). Lack of association between aspirin-triggered 15-hydroxy-eicosatetraenoic acid release and mast cell/eosinophil activation in nasal polyps from aspirin-sensitive patients. J. Investig. Allergol. Clin. Immunol. 21, 507–513. 22312933

[B74] Lundstrom K. (2009). An overview on GPCRs and drug discovery: structure-based drug design and structural biology on GPCRs. Methods Mol. Biol. 552, 51–66. 10.1007/978-1-60327-317-6_4 19513641 PMC7122359

[B75] Luu Q. Q. Moon J. Y. Lee D. H. Ban G. Y. Kim S. H. Park H. S. (2022). Role of thymus and activation-regulated chemokine in allergic asthma. J. Asthma Allergy 15, 157–167. 10.2147/JAA.S351720 PMC8828566 35153491

[B76] Ma C. Li Y. Ma J. Liu Y. Li Q. Niu S. (2011). Key role of 15-lipoxygenase/15-hydroxyeicosatetraenoic acid in pulmonary vascular remodeling and vascular angiogenesis associated with hypoxic pulmonary hypertension. Hypertension 58, 679–688. 10.1161/HYPERTENSIONAHA.111.171561 21859965

[B77] Marom Z. Shelhamer J. H. Sun F. Kaliner M. (1983). Human airway monohydroxyeicosatetraenoic acid generation and mucus release. J. Clin. Invest. 72, 122–127. 10.1172/jci110949 6308043 PMC1129167

[B78] Mastalerz L. Sanak M. Kumik J. Gawlewicz-Mroczka A. Celejewska-Wojcik N. Cmiel A. (2012). Exhaled eicosanoids following bronchial aspirin challenge in asthma patients with and without aspirin hypersensitivity: the pilot study. J. Allergy (Cairo) 2012, 696792. 10.1155/2012/696792 22291720 PMC3265180

[B79] Methner C. Liu L. Thompson A. Plascencia M. Chakravarty P. Kaul S. (2026). Deletion of GPR39 prevents pulmonary arterial hypertension by attenuating hypoxia-induced aberrant signaling. Available online at: https://www.biorxiv.org/cgi/content/short/2026.06.27.735008v1 (Accessed September 18, 2026).

[B80] Miyata J. Fukunaga K. Iwamoto R. Isobe Y. Niimi K. Takamiya R. (2013). Dysregulated synthesis of protectin D1 in eosinophils from patients with severe asthma. Allerg Y Clin. Immunol. 13, 353–360. 10.1016/j.jaci.2012.07.048 23006546

[B81] Miyata J. Sunata K. Sasaki H. Kawashima Y. Otsu Y. Onozato R. (2026). Multiomics insights into retinoic acid-mediated regulation of eosinophils in severe asthma. J. Allergy Clin. Immunol. 157, 531–539. 10.1016/j.jaci.2025.10.029 41207640

[B82] Nadel J. A. Conrad D. J. Ueki I. F. Schuster A. Sigal E. (1991). Immunocytochemical localization of arachidonate 15-lipoxygenase in erythrocytes, leukocytes, and airway cells. J. Clin. Invest. 87, 1139–1145. 10.1172/JCI115110 2010530 PMC295116

[B83] Nolin J. D. Murphy R. C. Gelb M. H. Altemeier W. A. Henderson W. R. Jr. Hallstrand T. S. (2019). Function of secreted phospholipase A(2) group-X in asthma and allergic disease. Biochim. Biophys. Acta. Mol. Cell Biol. Lipids 1864, 827–837. 10.1016/j.bbalip.2018.11.009 30529275 PMC6431270

[B84] Oppenheimer J. Hoyte F. C. L. Phipatanakul W. Silver J. Howarth P. Lugogo N. L. (2022). Allergic and eosinophilic asthma in the era of biomarkers and biologics: similarities, differences and misconceptions. Ann. Allergy Asthma Immunol. 129, 169–180. 10.1016/j.anai.2022.02.021 35272048

[B85] Pate C. A. Zahran H. S. (2024). The status of asthma in the United States. Prev. Chronic. Dis. 21, 240005. 10.5888/pcd21.240005 PMC11262137 39025120

[B86] Penn R. B. Bond R. A. Walker J. K. (2014). GPCRS and arrestins in airways: implications for asthma. Handb. Exp. Pharmacol. 219, 387–403. 10.1007/978-3-642-41199-1_20 24292841 PMC4429126

[B87] Pniewska E. Pawliczak R. (2013). The involvement of phospholipases A2 in asthma and chronic obstructive pulmonary disease. Mediat. Inflamm. 2013, 793505. 10.1155/2013/793505 24089590 PMC3780701

[B88] Poff C. D. Balazy M. (2004). Drugs that target lipoxygenases and leukotrienes as emerging therapies for asthma and cancer. Curr. Drug Targets Inflamm. Allergy. 3, 19–33. 10.2174/1568010043483917 15032639

[B89] Popovics P. Stewart A. J. (2011). GPR39: a Zn(2+)-activated G protein-coupled receptor that regulates pancreatic, gastrointestinal and neuronal functions. Cell. Mol. Life. Sci. 68, 85–95. 10.1007/s00018-010-0517-1 20812023 PMC11114682

[B90] Porpodis K. Tsiouprou I. Apostolopoulos A. Ntontsi P. Fouka E. Papakosta D. (2022). Eosinophilic asthma, phenotypes-endotypes and current biomarkers of choice. J. Per.s. Med. 12, 1093. 10.3390/jpm12071093 35887589 PMC9316404

[B91] Powell W. S. Rokach J. (2013). The eosinophil chemoattractant 5-oxo-EET and the OXE receptor. Prog. Lipid. Res. 52, 651–665. 10.1016/j.plipres.2013.09.001 24056189 PMC5710732

[B92] Prado C. M. Martins M. A. Tiberio I. F. (2011). Nitric oxide in asthma physiopathology. ISRN Allergy Online 2011, 832560. 10.5402/2011/832560 23724233 PMC3658695

[B93] Prevention C. D. C. (2024). Interactive Summary Health Statistics for Adults. Atlanta, GA: Centers for Disease Control.

[B94] Profita M. Sala A. Riccobono L. Paterno A. Mirabella A. Bonanno A. (2000). 15-Lipoxygenase expression and 15(S)-hydroxyeicoisatetraenoic acid release and reincorporation in induced sputum of asthmatic subjects. J. Allergy Clin. Immunol. 105, 711–716. 10.1067/mai.2000.105122 10756220

[B95] Rosenbaum D. M. Rasmussen S. G. Kobilka B. K. (2009). The structure and function of G-protein-coupled receptors. Nature 459, 356–363. 10.1038/nature08144 19458711 PMC3967846

[B96] Salari H. Chan-Yeung M. (1989). Release of 15-hydroxyeicosatetraenoic acid (15-HETE) and prostaglandin E2 (PGE2) by cultured human bronchial epithelial cells. Am. J. Respir. Cell. Mol. Biol. 1, 245–250. 10.1165/ajrcmb/1.3.245 2516453

[B97] Salari H. Schellenberg R. R. (1991). Stimulation of human airway epithelial cells by platelet activating factor (PAF) and arachidonic acid produces 15-hydroxyeicosatetraenoic acid (15-HETE) capable of contracting bronchial smooth muscle. Pulm. Pharmacol. 4, 1–7. 10.1016/0952-0600(91)90033-y 1804490

[B98] Schuliga M. (2015). NF-kappaB signaling in chronic inflammatory airway disease. Biomolecules 5, 1266–1283. 10.3390/biom5031266 26131974 PMC4598751

[B99] Schwenk U. Schröder J. M. (1995). Schroder JM. 5-Oxo-eicosanoids are potent eosinophil chemotactic factors. Functional characterization and structural requirements. J. Biol. Chem. 270, 15029–15036. 10.1074/jbc.270.25.15029 7797484

[B100] Shankar A. McAlees J. W. Lewkowich I. P. (2022). Modulation of IL-4/IL-13 cytokine signaling in the context of allergic disease. J. Allergy Clin. Immunol. 150, 266–276. 10.1016/j.jaci.2022.06.012 PMC9371363 35934680

[B101] Shannon V. R. Crouch E. C. Takahashi Y. Ueda N. Yamamoto S. Holtzman M. J. (1991). Related expression of arachidonate 12- and 15-lipoxygenases in animal and human lung tissue. Am. J. Physiol. 261 (6 Pt 1), L399–L405. 10.1152/ajplung.1991.261.6.L399 1767860

[B102] Shuaib Nasser S. M. Lee T. H. (2002). Products of 15-lipoxygenase: are they important in asthma? Clin. Exp. Allergy 32, 1540–1542. 10.1046/j.1365-2222.2002.01529.x 12569971

[B103] Singh N. K. Rao G. N. (2019). Emerging role of 12/15-Lipoxygenase (ALOX15) in human pathologies. Prog. Lipid Res. 73, 28–45. 10.1016/j.plipres.2018.11.001 30472260 PMC6338518

[B104] Skevaki C. Tafo P. Bahmer T. Abdo M. Watz H. Pedersen F. (2025). ALLIANCE study group as part of the German center for lung research. Differential expression of eicosanoid pathways after whole blood stimulation in asthma patients. World Allergy Organ. J. 18, 101047. 10.1016/j.waojou.2025.101047 PMC11999605 40235674

[B105] Skolnik N. S. Carnahan S. P. (2019). Primary care of asthma: new options for severe eosinophilic asthma. Curr. Med. Res. Opin. 35, 1309–1318. 10.1080/03007995.2019.1595966 30880484

[B106] Song Y. S. Kim M. S. Lee D. H. Oh D. K. Yoon D. Y. (2015). 15-Hydroxyeicosatetraenoic acid inhibits Phorbol-12-Myristate-13-Acetate-Induced MUC5AC expression in NCI-H292 respiratory epithelial cells. J. Microbiol. Biotechnol. 25, 589–597. 10.4014/jmb.1501.01052 25649981

[B107] Song B. Hu J. Chen S. Zhang Y. (2025). The mechanisms and therapeutic implications of PI3K signaling in airway inflammation and remodeling in asthma. Biologics 19, 73–86. 10.2147/btt.s497622 40070559 PMC11895685

[B108] Spanbroek R. Hildner M. Kohler A. Muller A. Zintl F. Kuhn H. (2001). IL-4 determines eicosanoid formation in dendritic cells by down-regulation of 5-lipoxygenase and up-regulation of 15-lipoxygenase 1 expression. Proc. Natl. Acad. Sci. U.S.A. 98, 5152–5157. 10.1073/pnas.091076998 11320251 PMC33179

[B109] Sridhar S. Liu H. Pham T. H. Damera G. Newbold P. (2019). Modulation of blood inflammatory markers by benralizumab in patients with eosinophilic airway diseases. Respir. Res. 20, 14. 10.1186/s12931-018-0968-8 30658649 PMC6339432

[B110] Stinson S. Amrani Y. Brightling C. (2014). D prostanoid receptor 2 (chemoattractant receptor–homologous molecule expressed on T_H_2 cells) protein expression in asthmatic patients and its effects on bronchial epithelial cells. J. Allergy Clin. Immunol. 135, 395–406.e7. 10.1016/j.jaci.2014.08.027 25312757 PMC4314591

[B111] Szatkowski P. Gielicz A. Stepien A. Hartwich P. Kacorzyk R. Plutecka H. (2024). Unique effect of aspirin on local 15-oxo-eicosatetraenoic acid synthesis in asthma patients with aspirin hypersensitivity. Clin. Transl. Allergy 14, e70004. 10.1002/clt2.70004 PMC11669626 39722441

[B112] Tufvesson E. Andersson C. Weidner J. Erjefalt J. S. Bjermer L. (2011). Inducible nitric oxide synthase expression is increased in the alveolar compartment of asthmatic patients. Allergy 72, 627–635. 10.1111/all.13052 27647044

[B113] Turi K. N. Michel C. R. Manke J. Doenges K. A. Reisdorph N. Bauer A. K. (2023). Multi-omics analysis of lung tissue demonstrates changes to lipid metabolism during allergic sensitization in mice. Metabolites 13, 406. 10.3390/metabo13030406 PMC10054742 36984845

[B114] Uehara S. Hirai K. Shirai T. Akamatsu T. Itoh K. (2025). PI3K pathway activation in severe asthma is linked to steroid insensitivity and adverse outcomes. J. Allergy Clin. Immunol. Glo. 4, 100439. 10.1016/j.jacig.2025.100439 PMC11928809 40125453

[B115] Wendell S. Fan H. Zhang C. (2020). G protein–coupled receptors in asthma therapy: pharmacology and drug action. Pharmacol. Rev. 72, 1–49. 10.1124/pr.118.016899 31767622 PMC6878000

[B116] Wenzel S. E. Schwartz L. B. Langmack E. L. Halliday J. L. Trudeau J. B. Gibbs R. L. (1999). Evidence that severe asthma can be divided pathologically into two inflammatory subtypes with distinct physiologic and clinical characteristics. Am. J. Respir. Crit. Care Med. 160, 1001–1008. 10.1164/ajrccm.160.3.9812110 10471631

[B117] World Health Organization (2026). Asthma. Available online at: https://www.who.int/news-room/fact-sheets/detail/asthma (Accessed September 18, 2026).

[B118] Wu X. Chen Y. Chen S. Lin Y. (2024). Blockade of cysteinyl leukotriene receptor 1 alleviates asthma by inhibiting bronchial epithelial cell apoptosis and activating the Nrf2 signaling pathway. Exp. Ther. Med. 29, 30. 10.3892/etm.2024.12780 PMC12141991 40486901

[B119] Yanni J. M. Foxwell M. H. Whitman L. L. Smith W. L. Nolan J. C. (1989). Effect of intravenously administered lipoxygenase metabolites on rat tracheal mucous gel layer thickness. Int. Arch. Allergy Appl. Immunol. 90, 307–309. 10.1159/000235043 2556352

[B120] Yoo E. J. Ojiaku C. A. Sunder K. Panettieri R. A. Jr (2017). Phosphoinositide 3-kinase in asthma: novel roles and therapeutic approaches. Am. J. Respir. Cell. Mol. Biol. 2056, 700–707. 10.1165/rcmb.2016-0308tr PMC5516292 27977296

[B121] Zhang J. Y. Wenzel S. E. (2007). Tissue and BAL based biomarkers in asthma. Immunol. Allergy Clin. North Am. 27, 623–632. 10.1016/j.iac.2007.09.003 17996580

[B122] Zhang Y. Saradna A. Ratan R. Ke X. Tu W. Do D. C. (2020). Rhoa/rho-kinases in asthma: from pathogenesis to therapeutic targets. Clin. Transl. Immunol. 9, e01134. 10.1002/cti2.1134 32355562 PMC7190398

[B123] Zhao J. Maskrey B. Balzar S. Chibana K. Mustovich A. Hu H. (2009). Interleukin-13-induced MUC5AC is regulated by 15-lipoxygenase 1 pathway in human bronchial epithelial cells. Am. J. Respir. Crit. Care Med. 179, 782–790. 10.1164/rccm.200811-1744OC 19218191 PMC2675565

[B124] Zhao J. O'Donnell V. B. Balzar S. St Croix C. M. Trudeau J. B. Wenzel S. E. (2011). 15-Lipoxygenase 1 interacts with phosphatidylethanolamine-binding protein to regulate MAPK signaling in human airway epithelial cells. Proc. Natl. Acad. Sci. U.S.A. 108, 14246–14251. 10.1073/pnas.1018075108 PMC3161579 21831839

[B125] Zhao J. Minami Y. Etling E. Coleman J. M. Lauder S. N. Tyrrell V. (2017). Preferential generation of 15-HETE-PE induced by IL-13 regulates goblet cell differentiation in human airway epithelial cells. Am. J. Respir. Cell. Mol. Biol. 57, 692–701. 10.1165/rcmb.2017-0031OC 28723225 PMC5765418

